# Dr. Myron H. Andrews

**Published:** 1904-07

**Authors:** 


Dr. Myron H. Andrews, of Detroit, a graduate of the University of Buffalo, 1847, died at his home June 3, 1904, from pneumonia, aged 87 years. He was a physician of prominence, having served as health physician of Detroit for three years (1888 to 1891), and as surgeon of the Michigan Central Railroad for several years.

